# *p*-Coumaric Acid Protects Human Lens Epithelial Cells against Oxidative Stress-Induced Apoptosis by MAPK Signaling

**DOI:** 10.1155/2018/8549052

**Published:** 2018-04-10

**Authors:** Jiao Peng, Ting-ting Zheng, Yue Liang, Li-fang Duan, Yao-dong Zhang, Li-Jun Wang, Guang-ming He, Hai-tao Xiao

**Affiliations:** ^1^Department of Pharmacy, Peking University Shenzhen Hospital, Shenzhen 518035, China; ^2^School of Pharmaceutical Sciences, Health Science Center, Shenzhen University, Shenzhen 518060, China; ^3^The Key Laboratory of Pharmacology and Druggability for Natural Medicines, Department of Education, Guizhou Medical University, Guiyang, Guizhou 550025, China; ^4^Shenzhen Key Laboratory for Drug Addiction and Medication Safety, Department of Ultrasound, Shenzhen-PKU-HKUST Medical Center, Peking University Shenzhen Hospital and Biomedical Research Institute, Shenzhen 518036, China

## Abstract

To protect against oxidative stress-induced apoptosis in lens epithelial cells is a potential strategy in preventing cataract formation. The present study aimed at studying the protective effect and underlying mechanisms of *p*-coumaric acid (*p*-CA) on hydrogen peroxide- (H_2_O_2_-) induced apoptosis in human lens epithelial (HLE) cells (SRA 01–04). Cells were pretreated with *p*-CA at a concentration of 3, 10, and 30 *μ*M before the treatment of H_2_O_2_ (275 *μ*M). Results showed that pretreatment with *p*-CA significantly protected against H_2_O_2_-induced cell death in a dose-dependent manner, as well as downregulating the expressions of both cleaved caspase-3 and cleaved caspase-9 in HLE cells. Moreover, *p*-CA also greatly suppressed H_2_O_2_-induced intracellular ROS production and mitochondrial membrane potential loss and elevated the activities of T-SOD, CAT, and GSH-Px of H_2_O_2_-treated cells. As well, *in vitro* study showed that *p*-CA also suppressed H_2_O_2_-induced phosphorylation of p-38, ERK, and JNK in HLE cells. These findings demonstrate that *p*-CA suppresses H_2_O_2_-induced HLE cell apoptosis through modulating MAPK signaling pathways and suggest that *p*-CA has a potential therapeutic role in the prevention of cataract.

## 1. Introduction

Age-related cataracts are the leading causes of blindness among the elderly population, affecting nearly 50% of 180 million visually impaired people in the world [1]. Although the surgery has proved effective for cataracts, it still exists in many risks and complications [2]. It is estimated that if the onset of clinical cataract can be delayed for 10 years, half of the cataract surgeries will not be necessary [3]. Thus, to delay or prevent cataract development would be important both for increasing the well-being of older adults and for reducing medical care costs. Oxidative stress caused by reactive oxygen species (ROS) has long been recognized as the major contributor to the formation of cataract, and hydrogen peroxide (H_2_O_2_) is the main intracellular ROS in the aqueous humor, which can activate multiple signaling events such as the activation of the caspases, the Bcl-2 family, the mitogen-activated protein kinases (MAPKs), and NF-*к*B pathways to induce lens epithelial cell (HLE) apoptosis and cause lens opacification, resulting in subsequent cataract development [4–7]. Therefore, to protect human lens epithelial cells from oxidative stress-induced apoptosis is an important strategy in preventing and delaying cataract formation.

*p*-Coumaric acid (*p*-CA) is a phenolic acid which is widely distributed in many plants and human diets such as cereals, fruits, and vegetables, possessing versatile medicinal activities including antioxidant, cardioprotective, antimelanogenic, antimutagenic, antiplatelet, anti-inflammatory, and immunomodulatory actions [8–13]. Previous studies reported that *p*-CA is effective to protect the cornea from UVB-induced oxidation damage *in vivo* and *in vitro* [14–16]. Other studies also proposed that *p*-CA could prevent cells from oxidative stress-induced apoptosis [17, 18]. However, the molecular mechanisms behind its protection against oxidative stress remain unknown. In our preliminary work, we screened the protective effects of natural compounds on H_2_O_2_-induced oxidative damage in human lens epithelial cells and found that *p*-CA exerts a potent protective effect on H_2_O_2_-induced oxidative stress in human lens epithelial cells (SRA 01–04). In the present study, we therefore investigated the effect of *p*-CA on H_2_O_2_-induced apoptosis in human lens epithelial (HLE) cells and its molecular mechanisms involved.

## 2. Materials and Methods

### 2.1. General

Fetal bovine serum (FBS), 0.25% trypsin, and DMEM/F12 medium were obtained from Gibco (Grand Island, NY, USA). Dimethyl sulfoxide (DMSO), H_2_O_2_, cocktail of protease inhibitors, and 3-(4,5-dimethylthiazol-2-yl)-2,5-diphenyltetrazolium bromide (MTT) were purchased from Sigma Chemical Co. (St. Louis, MO, USA). Quick start Bradford 1x dye reagent was from Bio-Rad Laboratories Inc. (Berkeley, California, USA). Anti-cleaved caspase-3, anti-cleaved caspase-9, anti-p-p38, anti-p-ERK, anti-p-JNK, anti-β-actin, U0126, LY2228820, and SP600125 were obtained from Cell Signal Technology (Beverly, MA, USA). The ECL detection kit was acquired from Amersham Pharmacia (Arlington Heights, IL, USA). The annexin V/FITC kit was purchased from eBioscience (Bender MedSystems GmbH, Vienna, Austria). T-SOD, CAT, and GSH-Px colorimetric activity assay kits were purchased from Jiancheng Bioengineering Institute (Nanjing, China). H2DCF-DA was obtained from Beyotime (Beyotime Institute of Biotechnology, Shanghai, China).

### 2.2. Cell Culture

Human lens epithelial (HLE) cells SRA 01/04 were purchased from the Chinese Academy of Medical Sciences (Beijing, China). The HLE cells were cultured as a monolayer in DMEM/F12 medium supplemented with 15% heat-inactivated FBS, penicillin (100 U/mL), and streptomycin (100 *μ*g/mL) and were maintained under standard cell culture conditions at 37°C in a humidified atmosphere of 5% CO_2_. The cells were routinely subcultured every 2-3 days.

### 2.3. Cell Viability

The cells were cultured in 96-well microplates with a density of 1 × 10^4^ cells/well and incubated with different concentrations of *p*-CA (0–100 *μ*M), respectively, with or without the exposure of H_2_O_2_. After 24 h incubation, the culture medium was removed and cell viability was measured using the MTT method as previously described [19].

### 2.4. Detection of Cell Apoptosis

The percentage of apoptotic cells was assayed using the annexin V/FITC apoptosis detection kit according to the manufacturer's directions. Briefly, cells were plated and incubated in 6-well plates at a density of 1 × 10^6^ cells/well. After exposure to H_2_O_2_ (275 *μ*M) and/or indicated concentrations of *p*-CA for 6 h, the cells were harvested by 0.25% trypsin and washed twice with ice-cold PBS. The cell pellets were resuspended in binding buffer (1x) prior to the addition of 5 *μ*L FITC-labeled annexin V and 10 *μ*L propidium iodide (PI) for 15 min at room temperature in the dark. The level of cell apoptosis was immediately analyzed on a flow cytometer (Becton Dickinson, San Jose, CA, USA).

### 2.5. Reactive Oxygen Species (ROS) Generation Assay

The intracellular ROS levels were evaluated using the ROS-specific fluorescent dye H2DCF-DA. After indicating treatments, cells were washed with PBS for three times and stained with H2DCF-DA, incubated at 37°C for 30 min in the darkness. Furthermore, cells were harvested and the generation of ROS was quantified by measuring the intracellular fluorescence intensity by a flow cytometer. The samples were then analyzed with FlowJo 7.6.1.

### 2.6. Measurement of Mitochondrial Membrane Potential (Δ*ψ*m) and the Activities of Antioxidant Enzymes

The mitochondrial membrane potential was tested using Rhodamine123 (Rh123) as previously described [20]. As well, T-SOD, CAT, and GSH-Px activities were all measured as previously described using a colorimetric assay kit according to the manufacturers' instructions [20].

### 2.7. Immunoblotting

Cells were incubated in 6-well plates at a density of 1 × 10^6^ cells/well. After exposure to H_2_O_2_ (275 *μ*M) and/or indicated concentrations of *p*-CA for 24 h, the cells were washed twice with ice-cold PBS and then lysed using RIPA buffer for 30 min on ice. Protein concentrations were quantified using the BCA protein assay kit [21]. And then, protein from each sample (50 *μ*g) was separated by 12% SDS-PAGE gels and transferred onto nitrocellulose membranes. After incubation with primary antibodies against cleaved caspase-3, cleaved caspase-9, p-p-38, p-ERK, p-JNK, and *β*-actin, followed by incubations with the appropriate secondary antibodies conjugated with HRP as our previously described method [22], the blots were detected by enhanced chemiluminescence (ECL), and then protein bands were quantified by densitometric analysis using Image-Pro Plus® software (Media Cybernetics Inc., USA).

### 2.8. Statistical Analysis

The data are presented as the mean value ± standard error of the mean (SEM). The difference between the mean values was evaluated using one-way ANOVA, followed by Duncan's multiple range tests. Comparisons were performed using GraphPad Prism 5.0 software (GraphPad Software Inc., San Diego, CA, USA). A *P* value < 0.05 was considered statistically significant.

## 3. Results and Discussion

**Figure 1:**
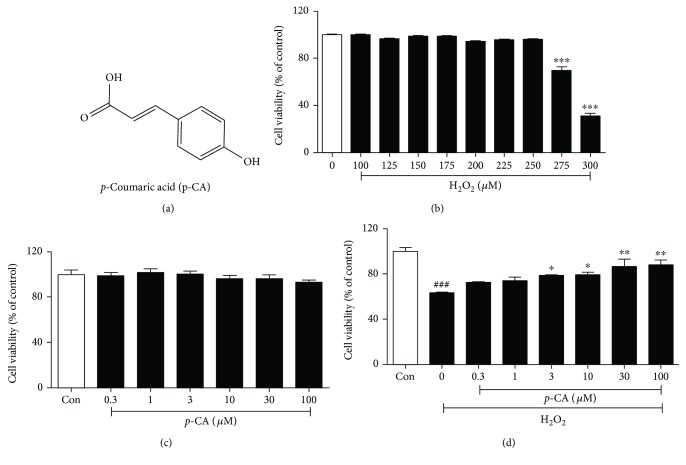
Effects of *p*-CA on cell viability of H_2_O_2_-induced cell death in HLE cells. (a) Structure of *p*-CA. (b) HLE cells were treated with a series of H_2_O_2_ concentrations (0–300 *μ*M) for 24 h. (c) HLE cells were incubated with different concentrations of *p*-CA (0–100 *μ*M) for 24 h. (d) HLE cells are preincubated with *p*-CA (0–100 *μ*M) for 2 h before 275 *μ*M H_2_O_2_ treated for 24 h. Cell viability was measured using the MTT method. Data are expressed as the mean ± SEM (*n* = 3). ^###^*P* < 0.001, compared with the untreated control group; ^∗^*P* < 0.05, ^∗∗^*P* < 0.01 and ^∗∗∗^*P* < 0.001, compared with the H_2_O_2_-treated group.

**Figure 2:**
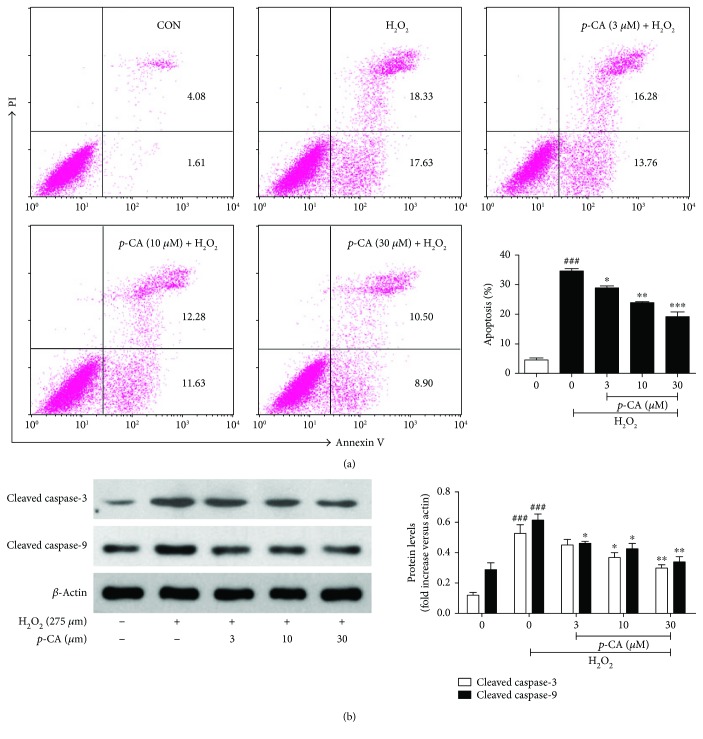
*p*-CA prevented H_2_O_2_-induced cell apoptosis in HLE cells. (a) HLE cells were pretreated with *p*-CA (3, 10, and 30 *μ*M) for 2 h before 275 *μ*M H_2_O_2_ treatment for 6 h. The apoptotic cells were detected by flow cytometry after staining with both FITC annexin V and PI. (b) HLE cells were pretreated with *p*-CA (3, 10, and 30 *μ*M) for 2 h before 275 *μ*M H_2_O_2_ treatment for 24 h. Cleaved caspase-3 and cleaved caspase-9 expressions were measured by Western blot. Data are expressed as the mean ± SEM (*n* = 3). ^###^*P* < 0.001, compared with the untreated control group; ^∗^*P* < 0.05, ^∗∗^*P* < 0.01, and ^∗∗∗^*P* < 0.001, compared with the H_2_O_2_-treated group.

It is well-known that the apoptosis of lens epithelial cells plays a vital role in cataract formation, and oxidative stress induced by ROS such as O_2_^−^ and H_2_O_2_ has been recognized as an important mediator to induce apoptosis of lens epithelial cells [3, 5, 6]. Therefore, in the study, H_2_O_2_ was used to induce oxidative injury of lens epithelial cells. After H_2_O_2_ exposure, the cells showed a high cell apoptosis rate and 275 *μ*M H_2_O_2_ was set as the working concentration (Figure 1(b)). In parallel, HLE cells were treated with different concentrations of *p-*CA (0, 0.3, 1, 3, 10, 30, and 100 *μ*M) for 24 h and no significant cytotoxicity was observed (Figure 1(c)). And then, the dose response of *p*-CA was assayed on H_2_O_2_-treated HLE cells, and the result showed that pretreatment with *p*-CA (3–100 *μ*M) significantly prevented H_2_O_2_-induced cell death in a dose-dependent manner (Figure 1(d)). To quantitatively examine the protective potential of *p*-CA against H_2_O_2_-induced cell death in HLE cells, the percentage of apoptotic cells was detected by the annexin V/PI double staining method. As shown in Figure 2(a), the percentage of total apoptotic cells (the sum of early-stage apoptotic cells (annexin V^+^/PI^−^ cells) and late-stage apoptotic cells (annexin V^+^/PI^+^)) was significantly increased after the exposure of 275 *μ*M H_2_O. Compared to the cells treated with H_2_O_2_ alone, pretreatment with *p*-CA (3, 10, and 30 *μ*M) resulted in a significant decrease of apoptotic cells in the total population of cells in a dose-dependent manner. Caspases are the main initiators and executioners of apoptosis [23, 24]. As one of the key effectors, caspase-3 is activated through cleavage by caspase-9 and involved in the mitochondria-mediated pathway. We thus examined the expressions of cleaved caspase-3 and cleaved caspase-9 using Western blot. As shown in Figure 2(b), the expressions of cleaved caspase-3 and cleaved caspase-9 were markedly upregulated after H_2_O_2_ treatment. In contrast, pretreatment with *p*-CA (3, 10, and 30 *μ*M) significantly inhibited the upregulation of cleaved caspase-3 and cleaved caspase-9. Collectively, these results suggested that *p*-CA exhibits a potent protective effect against H_2_O_2_-induced apoptosis in HLE cells.

**Figure 3:**
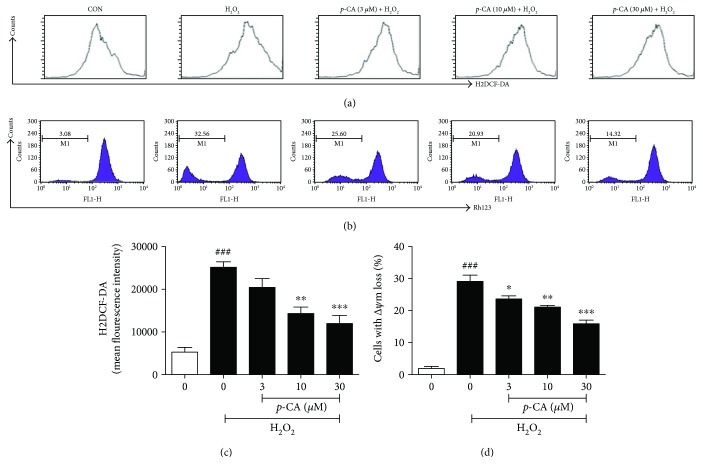
*p*-CA suppressed intracellular ROS production and mitochondrial membrane potential loss in H_2_O_2_-treated HLE cells. (a) HLE cells were pretreated with *p*-CA (3, 10, and 30 *μ*M) for 2 h before 275 *μ*M H_2_O_2_ treatment for 6 h. After staining with H2DCF-DA, cells were incubated at 37°C for 30 min in the darkness and then detected by flow cytometry. (b) HLE cells were preincubated with *p*-CA (3, 10, and 30 *μ*M) for 2 h before 275 *μ*M H_2_O_2_ treatment for 6 h. Cells were incubated with Rh123 for 30 min at 37°C, and then the fluorescent intensity was analyzed using flow cytometry. Data are expressed as the mean ± SEM (*n* = 3). ^###^*P* < 0.001, compared with the untreated control group; ^∗^*P* < 0.05, ^∗∗^*P* < 0.01, and ^∗∗∗^*P* < 0.001, compared with the H_2_O_2_-treated group.

**Figure 4:**
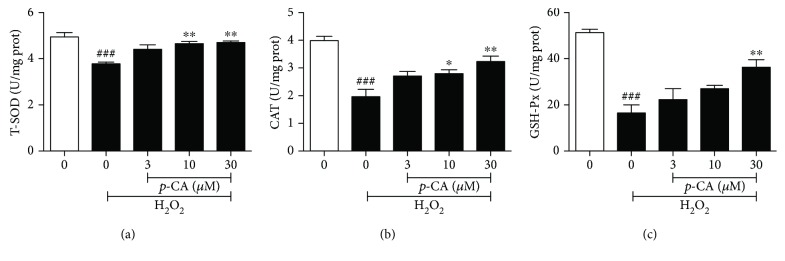
*p*-CA enhanced the activities of T-SOD, CAT, and GSH-Px in H_2_O_2_-treated HLE cells. HLE cells were pretreated on *p*-CA (3, 10, and 30 *μ*M) for 2 h before 275 *μ*M H_2_O_2_ treatment for 24 h. The activities of T-SOD (a), CAT (b), and GSH-Px (c) were measured by commercial assay kits. Data were expressed as the mean ± SEM (*n* = 3). ^###^*P* < 0.001, compared with the untreated control group; ^∗^*P* < 0.05 and ^∗∗^*p* < 0.01, compared with the H_2_O_2_-treated group.

Oxidative stress can be defined as the imbalance between prooxidant/antioxidant that can occur as a result of an increase in free radical production and/or a decrease in radical scavenging capability of antioxidant defense systems. We therefore detected the production of intracellular free radical ROS in H_2_O_2_-induced HLE cells, as shown in Figure 3(a). When the cells were exposed to H_2_O_2_, the generation of ROS increased significantly, whereas pretreatment with *p*-CA (3, 10, and 30 *μ*M) significantly inhibited H_2_O_2_-induced production of intracellular ROS in a dose-dependent manner. The mitochondria are the major location to produce intracellular ROS, and ROS overload would result in lysosomal leakage through the mitochondrial outer membrane and mitochondrial dysfunction. A significant reduction of mitochondrial membrane potential (Δ*ψ*m) is a symbolic feature of early apoptosis. We further determined the loss of Δ*ψ*m in H_2_O_2_-induced HLE cells. As shown in Figure 3(b), exposure of HLE cells to H_2_O_2_ for 6 h could significantly increase the percentage of cells with Δ*ψ*m loss, which is up to 29.0%, and pretreatment with *p*-CA (3, 10, and 30 *μ*M) could significantly reduce the percentage of cells with Δ*ψ*m loss to 23.7%, 21.2%, and 15.8% in a dose-dependent manner. In parallel, we also detected the radical scavenging capability of antioxidant defense systems of HLE cells, such as the activities of T-SOD, GSH-Px, and CAT enzymes. Compared to the untreated control, H_2_O_2_ decreased the activities of T-SOD, CAT, and GSH-Px near 2-fold. In H_2_O_2_-treated HLE cells, pretreatment with *p*-CA (3, 10, and 30 *μ*M) could significantly enhance the activities of T-SOD, CAT, and GSH-Px in dose-dependent manners as shown in Figure 4. These results indicated that *p*-CA possesses potent antioxidant activities against H_2_O_2_-induced impairment in HLE cells.

**Figure 5:**
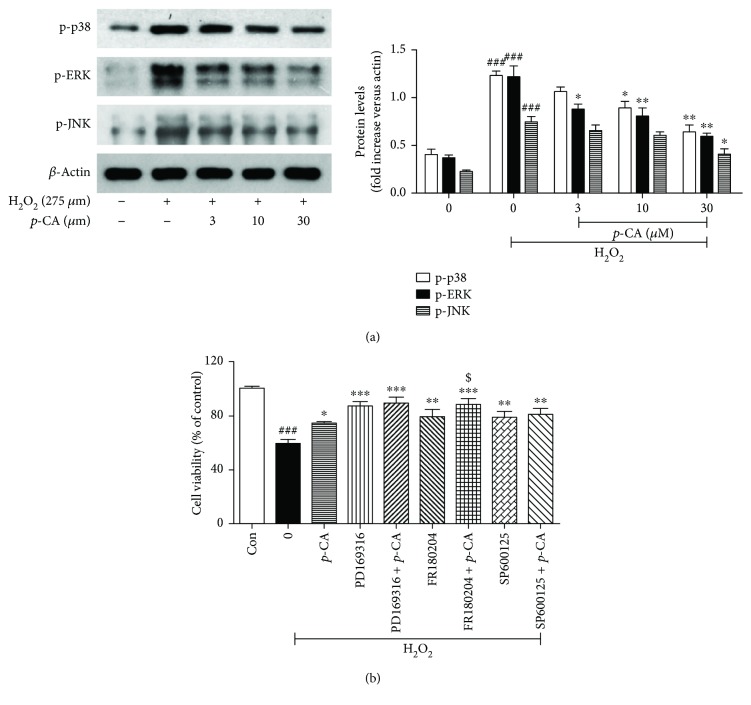
*p*-CA suppressed MAPK signals in H_2_O_2_-treated HLE cells. (a) HLE cells were pretreated on *p*-CA (3, 10, and 30 *μ*M) for 2 h before 275 *μ*M H_2_O_2_ treatment for 24 h. Cells were lysed, and then p-p-38, p-ERK, and p-JNK expressions were measured using Western blot. (b) HLE cells were pretreated on *p*-CA (10 *μ*M) or ERK inhibitor (FR180204, 1.25 *μ*M) or p-38 inhibitor (PD169316, 1.25 *μ*M) or JNK inhibitor (SP600125, 1.25 *μ*M) or a combination of both *p*-CA and SP600125 or PD169316 and FR180204 for 2 h before 275 *μ*M H_2_O_2_ treatment for 24 h; then the cell viability was measured using the MTT method. Data are expressed as the mean ± SEM (*n* = 3). ^###^*P* < 0.001, compared with the untreated control group; ^∗^*P* < 0.05, ^∗∗^*P* < 0.01 and ^∗∗∗^*P* < 0.001, compared with the H_2_O_2_-treated group; and ^$^*P* < 0.05, compared with the H_2_O_2_ + *p*-CA group using Student's *t*-test.

To explore the molecular mechanisms involved in the protective effects of *p*-CA against H_2_O_2_-induced apoptosis, we evaluated the possible influence of this compound on the expression of mitogen-activated protein kinases (MAPKs), namely, ERK, JNK, and p38, which play important roles in the regulation of intracellular metabolism and responding to external stress [6]. Several previous investigations reported that *p*-CA reduced the oxidative stress of various cells via the inhibition of the MAPK signaling cascade [25–27]. Our data showed that (Figure 5(a)) incubation with H_2_O_2_ led to a massive increase of p-p38, p-ERK, and p-JNK. As expected, increased p-p38, p-JNK, and p-ERK were significantly rescued by *p*-CA in a dose-dependent manner. To further confirm the role of the MAPK signaling pathway to mediate the inhibitory effect of *p*-CA on H_2_O_2_-induced apoptosis in HLE cells, we have detected the cell viability of cells exposed to H_2_O_2_ with the ERK inhibitor (U0126) or p-38 inhibitor (LY2228820) or JNK inhibitor (SP600125), respectively. As shown in Figure 5(b), compared to cells treated with *p*-CA alone in the presence of H_2_O_2_, HLE cells treated with *p*-CA combined with LY2228820 or U0126 or SP600125 in the exposure of H_2_O_2_ exhibited a much higher cell viability, which displayed the protective effect of *p*-CA on H_2_O_2_-induced apoptosis in HLE cells via affecting MAPK signaling pathways.

## 4. Conclusion

In conclusion, the data from this study demonstrate that *p*-CA, a natural compound, could protect HLE cells from oxidative stress-induced apoptosis through modulating MAPK signaling pathways. Our findings suggest a potential use of *p*-CA in the prevention of cataractogenesis.
